# Examining Individual- and Community-Level Factors Affecting Skilled Delivery Care among Women Who Received Adequate Antenatal Care in Ethiopia: Using Multilevel Analysis

**DOI:** 10.1155/2020/2130585

**Published:** 2020-09-28

**Authors:** Eshetu E. Chaka

**Affiliations:** Department of Public Health, College of Medicine and Health Sciences, Ambo University, Ambo, Ethiopia

## Abstract

**Introduction:**

Maternal mortality continues to be a major public health and development challenge in Africa even after the permissible commitment of the international community. Although the use of skilled delivery care is the key intervention and is effective to lower maternal mortality rates, it is still at a lower proportion. The study is aimed at investigating the individual- and community level factors affecting the use of skilled delivery care among those women who had received adequate antenatal care.

**Materials and Methods:**

Data were extracted from the 2016 Ethiopian Demographic and Health Survey on women aged 15-49 years and gave birth within five years prior to the survey (*N* = 957). Multilevel logistic regression model with two levels were fitted to assess the influence of the individual- and community-level factors on the use of skilled delivery care.

**Results:**

Women who were exposed to media were more likely to use skilled delivery care (OR = 1.81; 95% CI: 1.20-2.74). Having six or more birth order (OR = 0.33; 95% CI: 0.16-0.69) and residing in rural areas (OR = 0.40; 95% CI: 0.21-0.79) were associated with less likelihood use of skilled delivery care. Attaining primary and secondary educational level, being older women, being from the richest household, and having a urine test during antenatal visits were significantly associated with the use of skilled delivery care. The value of intraclass correlation coefficient supported a significant community-level effect on the likelihood of using skilled delivery care.

**Conclusions:**

Factors operating both at the individual level and community level were found significantly associated with the use of skilled delivery care in Ethiopia. A considerable variation at community level accounts for the difference in the use of skilled delivery level.

## 1. Introduction

Maternal mortality continues to be a major public health and development challenge in Africa compared to performance in the rest of the world even after the permissible commitment of the international community during MDG [1]. Increasing access to skilled delivery care during delivery is the key intervention and has proved effective to lower maternal mortality rates globally and in Sub-Saharan Africa [2]. Moreover, the World Health Organization recommends that all women should be attended by a skilled birth attendant during childbirth [3]. However, only 17 percent of pregnant women were attended by a skilled birth attendant in Ethiopia [4]. A skilled health professional can administer interventions to control and manage major life-threatening complications, such as hemorrhage or refer the patient to the next higher level health care facility [3].

Many studies have been done on the utilization and factors associated with skilled delivery care in Ethiopia [5–10]. Nevertheless, these studies were emphasized on individual and household factors and overlooking the contribution of community factors on the utilization of skilled delivery care during delivery. Ignoring the influence of community factors might underestimate the importance of considering them during planning appropriate interventions in scaling up safe motherhood in the country.

Existing literatures showed that the influence of individual- and household-level factors on the use of skilled delivery care vary across geographic and social settings. For example, the women with similar socioeconomic characteristics may have different healthcare-seeking behavior depending on whether they live in one community or another so the contextual factors that cluster individuals within communities have become a core notion of social epidemiology [11, 12]. Moreover, a study in Tanzania also showed that poorer households in wealthy regions are found to be better off in maternal health care utilization outcomes than poorer households in poorer regions [13]. Recently, many studies have reported that contextual/community factors are a key determine of maternal health utilization [14–17].

In the case of Ethiopia, there is only one study that tries to consider contextual-level factors associated with skilled delivery care during delivery among women with live birth using the outdated 2011 EDHS [1]. The main research question is why at least those women who had adequate antenatal care (begun first antenatal care within the first trimester, had received at least four antenatal care visits, and had antenatal care from the skilled provider) did not have delivery care by skilled birth providers. This study is aimed at assessing factors associated with the use of skilled delivery care during delivery including unexplored contextual factors in a nationally representative sample using the recent 2016 Ethiopia Demographic and Health Survey (EDHS).

## 2. Materials and Methods

### 2.1. Data and Study Design

The data for this cross-sectional study were extracted from the 2016 Ethiopia Demographic Health Survey (EDHS), a nationally representative household survey. The survey used a stratified two-stage cluster sampling design. Using the 2007 Ethiopian Population and Housing Census, 645 primary sampling units (PSUs) or clusters were selected using probability proportional at the first stage. In the second stage, a sample of the household was drawn from a listing of households in each of the sampled clusters. The survey included a nationally representative sample of 16,650 households and a total of 7,590 women with live births.

All women aged between 15 and 49 years and residents of Ethiopia were eligible for an interview in the survey. This study was restricted to 957 women who had received adequate antenatal care: four or more antenatal visits, began a first antenatal visit within the first trimester, and had skilled antenatal care and gave birth in the last 5 years preceding the survey. For women who had more than one live birth, only the most recent live birth was considered in this study to get information about the recent birth. Questionnaires were used to collect information in three areas: the household, the women's, and the men's questionnaires.

### 2.2. Outcome Variables

The outcome variable of this study is the use of skilled delivery care in a health care facility. Skilled delivery care in a health care facility is a binary variable and coded with a value of 1 if doctors, nurses, midwives, or health officers assisted the childbirth and a value of 0 otherwise.

### 2.3. Exposure Variables

Individual-level factors include the age of mothers at last birth, mother's education level, mother's employment, marital status, household wealth quintile, religion, exposure to media, sex of household, informed about pregnancy complication during pregnancy, blood pressure measured during ANC, a blood sample taken during ANC, urine sample taken during ANC, parity, birth order, and transportation problem to the delivery place. The household wealth index is calculated in the survey using the respondent's household assets by principal component analysis (PCA) and is coded as 1 for “poorest,” 2 for “poorer,” 3 for “middle,” 4 for “richer,” and 5 for “richest.” Community-level factors include the regional state and type of residence.

### 2.4. Data Analyses

A descriptive analysis is done for all the individual- and community-level factors to examine their distribution and association with the use of skilled delivery care. Multilevel logistic regression was employed to examine the effect of the individual and community characteristics on the use of skilled delivery care. The usage of the multilevel modeling technique was justified by the hierarchical structure of the survey data, where women are nested within the primary sampling unit or cluster or community. The clustering of observations within the same cluster violates the ordinary logistic regression model independence assumption. Thus, a multilevel modeling strategy accounts for the hierarchical nature of the data and corrects the estimated standard error. A two-level multilevel logistic regression model was performed to test the effect of each independent variable on the use of skilled delivery care in a health facility. Three sequential models were fitted. Firstly, model-0 or the intercept-only model was fitted without explanatory variables. Secondly, model-1 was fitted with only individual and household variables. Thirdly, model-2 was fitted with only community-level variables. Lastly, model-3 was fitted with a community-level variable in addition to individual and household variables. The multilevel binary logistic regression model equation is written as follows:

(1) $$\begin{array}{c} \text{Logit}\left(P_{ij}\right)=\beta 0+\beta_{1}I+\beta_{2}C+u_{j}, \end{array}$$

where *P*_*ij*_ is the probability of skilled delivery care for women *i* nested in the cluster *j*, the *β*'s are the fixed coefficients, *I* and *C* refer to the individual- and community-level-independent variables, respectively, and *u*_*j*_ indicate the random effects for the *j*^th^ cluster.

Measures of association (fixed effects) are measured using the odds ratio (OR) along with 95% confidence intervals (CI).

A measure of variation (random effects) is measured using the variance partition coefficient (VPC) and proportional change in variance (PCV). The variance partition coefficient or the variance coefficient (ICC) was assessed as shown:

(2) $$\begin{array}{c} \text{ICC}=\frac{{\sigma u}^{2}}{{\sigma u}^{2}+\pi 2/3}, \end{array}$$

where *π*2/3 denotes the variance between mothers in the same cluster (individual level) and *σ*^2^*u* is the variance between clusters (community-level variance). It measures the extent of the variance explained at the community level [18]. The entire analysis was done using STATA version 14.

## 3. Results

### 3.1. Description of Skilled Delivery Care Use by Background Characteristics

Table 1 presents the percentage distribution of the use of skilled delivery care by the participant's background characteristics. The percentage of skilled delivery care use was fairly equivalent across maternal age at last birth, 66% was aged 15-24 years, 67% was aged 25-34 years, and 57% was aged 35-49 years. The utilization of skilled delivery care was higher among the women who have attended primary and secondary or higher school as compared to those with no formal education.

Women who reside in urban areas, formally employed, from the richest household, reported Orthodox Christian affiliation, had first birth order, exposed to mass media, and had been informed about pregnancy complications during antenatal care visit were the advantaged group to use skilled delivery care. Moreover, those women who had blood pressure measured, had a blood sample tested, and had a urine test during antenatal care visits were more likely to use skilled delivery care as compared to their counterparts.

### 3.2. Multivariable Multilevel Logistic Analysis

Table 2 presents the result of multivariable multilevel binary logistic regression analysis. Older women were significantly more likely to use skilled delivery care than women age 15-24 years in model-3, the best-fitted model as it has the lowest Akaike Information Criterion statistics. Mother's educational level is found to have a significant association with skilled delivery care. Women who attended primary and secondary or higher education were significantly more likely to use skilled delivery care than women not have an education. Among household wealth quintile, only those women from the richest household were found to have a significant association with skilled birth delivery care. Living in the richest household increases the odds of skilled delivery care use than living in the poorest household. Women with six or more birth order were 66% less likely to use skilled delivery care in a health facility than women with the first birth order. Women who are exposed to radio or television or newspaper at least once per week have a higher likelihood of using skilled delivery care than those women not exposed to the media.

The odds of using skilled delivery care was higher among women who perceived that distance to a health facility to get health care is not a big problem compared to those who perceived it as a big problem.

As shown in model-3 of Table 2 community-level variables have a significant association with the use of skilled delivery care. Women who reside in rural areas were 60% less likely to use skilled delivery care compared to those residing in urban areas.

### 3.3. Random Effect Parameter and Model Fitness Statistics

Table 3 shows the intracluster correlation and model fit statistics. The findings in model-0 show that 53% of the total variance in the use of skilled delivery care is attributable to the community-level differences. The use of skilled delivery care is clustered significantly by the community. The intracluster correlation coefficients for the use of skilled delivery care reduced by more than half when individual-level variables were added into model-1. When the model was fitted with only community-level variables, 19.1% of the community-level variance in the use of skilled delivery care across clusters was explained.

However, when the community-level factors and individual-level factors were entered in model-3 simultaneously, the estimate of the intracluster correlation for the use of skilled delivery care shows nearly no change when compared with model-1. Although the percentage of the intracluster correlations reduced substantially, there is still a statistically significant community-level variance across each model that remains unexplained. This finding indicates that there are unobserved variables not included in this study that is related to the clustering in the use of skilled delivery care.

## 4. Discussion

The finding showed that near to two-thirds of women 617 (64.5%; 95% CI: 61.5-67.5) use the skilled delivery care in health facilities among women who had received adequate antenatal care in Ethiopia.

In model-3 in which individual- and community level factors were entered simultaneously, there is a significant association between maternal age at last birth and the use of skilled delivery care.

The finding revealed that the odds of using skilled delivery care are higher among older mothers compared to younger mothers. This might due to that older woman have a better awareness of availability and accessibility of health care services, and they have maternal health services experience than younger women. For instance, women in the age group 35-49 at last birth were twice (OR = 2.07; 95% CI: 1.07, 4.03) more likely to use skilled delivery care than those in the age group 15-24. This finding is supported by the results of the previous studies elsewhere. For example, older women were more likely to use delivery care and studies argued that older age women have better maternal health care experience and awareness of the availability of maternal health care service [1, 10, 16, 19].

The odds of using skilled delivery care decreased consistently as the order of birth increases among women who had received adequate antenatal care. That means women with higher birth orders are less likely to use skilled delivery care either because of having fewer resources due to large family size or their maternal health care experiences as mentioned in the previous study [10, 16, 20].

Women's educational attainment was significantly associated with the use of skilled delivery care. Women who had attained primary and secondary or above educational levels had higher odds of using skilled delivery care than those with no education. Moreover, the likelihood of using skilled delivery cares increased positively with the educational level of women. This result was consistent with the previous studies [21–24]. The previous studies explained that educated mothers have better access to health problem information, a high level of health literacy, awareness to overcome cultural barriers, and good power of decision making. Attending a higher education level empowers women to control health care and to encourage to access quality maternal health care.

The likelihood of using skilled delivery care was higher among women from the richest household than those from the poorest household. This finding is in line with other studies elsewhere [19, 25].

Moreover, the study also found that access/exposure to media had a significant association with the use of skilled delivery care. Having exposure to media influences the knowledge of women about health care needs during delivery and the availability and accessibility of such services [1, 24].

The study result showed a significant association between women's residence and the use of skilled delivery care. For example, women residing in rural areas were less likely to use skilled delivery care as compared with those residing in urban areas. This finding is consistent with studies done elsewhere. Scattered nature of the rural area, limited supply of health facilities, a far distance from health facilities, and difficulty accessing information and media in rural areas are some of the reasons mentioned [1, 16, 24]. However, a region, where women reside, was not found significantly associated with the use of skilled delivery.

## 5. Conclusions

At the individual level, being in an older age group, residing in the richest household, attaining a primary and secondary education level, having exposure to the media, and had a urine test during antenatal care were significantly associated with the use of skilled delivery care. Moreover, having six or more birth order was inversely associated with skilled delivery care. At the community level, residing in rural areas was significantly associated with the use of skilled delivery. Factors affecting the use of skilled delivery care operated both at the individual and community levels. The study demonstrated that there is a significant variation in the use of skilled delivery care across the community. Thus, any interventions intended to increase the use of skilled delivery care should consider community-level factors at large. This study also indicated the need to contextualize the strategies and explore the factors responsible for the unexplained variance in the use of skilled delivery care.

## Figures and Tables

**Table 1:** Distribution of skilled delivery care in a health facility by background characteristics.

Background characteristics	Yes (%)	No (%)	*P* value
Maternal age at last birth	**957**		0.2062
15-24	65.7	34.3	
25-34	66.8	33.2	
35-49	56.7	43.3	
Current marital status			0.6386
Never/widowed/divorced/separated	67.7	33.3	
Currently married or living together	64.2	35.8	
Mother's employment			0.0001
Unemployed	60.1	39.9	
Formal employment	88.3	11.7	
Agricultural employment	59.2	40.8	
Unskilled manual workers	69.1	30.9	
Type of place of residence			0.0001
Urban	91.8	8.2	
Rural	48.6	51.4	
Mother's educational attainment			0.0001
No education	44.2	55.8	
Primary	67.3	32.7	
Secondary or higher	92.4	7.6	
Household wealth index			0.0001
Poorest	41.2	58.8	
Poor	43.7	56.3	
Middle	47.8	52.2	
Rich	50.7	49.3	
Richest	91.8	8.2	
Region			0.0001
Oromia	58.6	41.4	
SNNP	54.7	45.3	
Amhara	53.1	46.9	
Addis Ababa & Dire Dawa	94.5	5.5	
Tigray	79.2	20.8	
Others	63.5	36.5	
Religion			0.009
Orthodox	70.3	29.7	
Protestant & others	52.2	47.8	
Muslim	57.2	42.8	
Parity (number of children)			0.0001
1 = at most one	75.2	24.8	
2	74.9	25.1	
3-4	55.1	44.9	
5 or more	44.2	55.8	
Birth order			0.0001
First	77.4	22.6	
2-3	68.67	31.3	
4-5	54.4	45.6	
6+	36.7	63.3	
Wanted pregnancy when became pregnant			0.9213
Yes, then	65.0	35.0	
Yes, later	62.8	37.2	
No more	62.3	37.5	
Told about pregnancy complications			0.0013
No	56.2	43.8	
Yes	70.6	29.4	
Exposed to media			0.0001
No	43.4	56.6	
Yes	80.7	19.3	
Sex of household head			0.0006
Male	60.8	39.2	
Female	80.0	20.0	
Getting money needed for self-treatment			0.0001
A big problem	52.6	47.4	
Not a big problem	73.6	26.4	
Distance to a health facility to get medical help			0.0001
A big problem	50.3	49.7	
Not a big problem	73.7	26.3	
Getting permission to go to a health facility			0.0011
A big problem	50.4	49.6	
Not a big problem	69.0	31.0	
Blood pressure checked at least once during ANC			0.0003
No	41.0	59.0	
Yes	67.5	32.5	
A urine test conducted during ANC			0.0001
No	34.0	66.0	
Yes	68.0	32.0	
A blood test conducted during ANC			0.0001
No	33.4	66.6	
Yes	68.2	31.8	
Received at least one tetanus injection during ANC			0.4333
No	60.4	39.6	
Yes	65.5	34.5	

**Table 2:** Multivariable multilevel logistic regression analysis of individual-level and community-level factors associated with the use of skilled delivery care.

Background characteristics	Model0	Model-1	Model-2	Model-3
Maternal age at last birth				
15-24		1.0	—	1.0
25-34		1.22 (0.75, 1.96)	—	1.22 (0.75, 1.96)
35-49		2.14 (1.10, 4.17)^∗^	—	2.07 (1.07, 4.03)^∗^
Mother's educational attainment				
No education		1.0	—	1.0
Primary		1.69 (1.12, 2.57)^∗^	—	1.71 (1.13, 2.59)^∗^
Secondary or higher		4.25 (2.32, 7.77)^∗∗^	—	4.08 (2.23, 7.45)^∗∗∗^
Household wealth index				
Poorest		1.0	—	1.0
Poor		1.32 (0.74, 2.37)	—	1.34 (0.75, 2.39)
Middle		1.41 (0.76, 2.58)	—	1.40 (0.77, 2.55)
Rich		1.03 (0.55, 1.91)	—	0.99 (0.54, 1.84)
Richest		5.27 (2.82, 9.83)^∗∗^	—	2.60 (1.22, 5.56)^∗^
Birth order				
First		1.0	—	1.0
2-3		0.80 (0.49, 1.31)	—	0.79 (0.48, 1.29)
4-5		0.68 (0.37, 1.27)	—	0.66 (0.35, 1.21)
6+		0.35 (0.17, 0.74)^∗∗^	—	0.33 (0.16, 0.69)^∗∗^
Exposed to media				
No		1.0	—	1.0
Yes		1.83 (1.21, 2.78)^∗∗^	—	1.81 (1.20, 2.74)^∗^
Getting money				
A big problem		1.0	—	—
Not a big problem		1.46 (1.00, 2.12)^∗^	—	—
Blood pressure checked at least once during ANC				
No		1.0	—	1.0
Yes		1.64 (0.90, 2.98)	—	1.61 (0.88, 2.92)
A urine test conducted during an antenatal visit				
No		1.0	—	1.0
Yes		2.14 (1.15, 3.99)^∗^	—	2.03 (1.09, 3.77)^∗^
Distance to a health facility to get medical help				
A big problem		1.0	—	—
Not a big problem		1.59 (1.10, 2.32)^∗^	—	—
Type of place of residence				
Urban		—	1.0	1.0
Rural		—	0.03 (0.011, 0.07)	0.40 (0.21, 0.79)^∗∗^
Region		—		
Oromia		—	1.0	1.0
SNNP		—	1.10 (0.44, 2.67)	1.20 (0.48, 1.45)
Amhara		—	0.77 (0.33, 1.81)	0.70 (0.43, 1.85)
AA & DW		—	3.64 (0.94, 14.10)	2.04 (0.90, 9.00)
Tigray		—	5.40 (2.06, 14.14)^∗^	3.21(0.13, 8.10)^∗^
Others		—	0.78 (0.24, 2.51)	0.77 (0.24, 2.51)

**Table 3:** Random effect parameters and model fit statistics in the use of skilled delivery care in a health facility, Ethiopia.

Random effects	Model-0	Model-1	Model-2	Model-3
Community level variance (SE)	3.756 (0.846)^∗∗∗^	0.971 (0.356)^∗∗^	3.04 (0.673)^∗∗^	0.933 (0.341)^∗∗^
ICC (VPC) (%)	53.3	22.7	48.0	22.1
Explained variance or PCV (%)	Reference	74.0	19.1	75.2
Model fit statistics				
Deviance	1318.86	1050.80	1070.14	1043.18
AIC	1322.86	1086.81	1086.14	1079.19
BIC	1333.07	1178.71	1127.71	1171.09

SE: standard error: ICC: intracluster correlation; VPC: variance partition coefficient; PCV: proportional change in variance.

## Data Availability

The data used to support the findings of this study are available from the Measure DHS project upon request.
